# Fine excipient materials in carrier-based dry powder inhalation formulations: The interplay of particle size and concentration effects

**DOI:** 10.1016/j.ijpx.2024.100251

**Published:** 2024-05-01

**Authors:** Mustafa M.A. Elsayed, Iman M. Alfagih, Katrina Brockbank, Fawaz Alheibshy, Alhassan H. Aodah, Raisuddin Ali, Khaled Almansour, Ahmed O. Shalash

**Affiliations:** a Department of Pharmaceutics, College of Pharmacy, University of Ha'il, Ha'il, Saudi Arabia; b Department of Pharmaceutics, Faculty of Pharmacy, Alexandria University, Alexandria, Egypt; c Department of Pharmaceutics, College of Pharmacy, King Saud University, Riyadh, Saudi Arabia; d Freeman Technology Ltd., Tewkesbury, United Kingdom; e Advanced Diagnostics and Therapeutics Institute, Health Sector, King Abdulaziz City for Science and Technology (KACST), Riyadh, Saudi Arabia; f School of Chemistry and Molecular Biosciences, The University of Queensland, St. Lucia, Queensland, Australia

**Keywords:** Dry powder inhalation, Carrier, Fine excipient material, Quality-by-design (QbD), Critical material attributes, Powder rheology, Mixing energy

## Abstract

The contributions of fine excipient materials to drug dispersibility from carrier-based dry powder inhalation (DPI) formulations are well recognized, although they are not completely understood. To improve the understanding of these contributions, we investigated the influences of the particle size of the fine excipient materials on characteristics of carrier-based DPI formulations. We studied two particle size grades of silica microspheres, with volume median diameters of 3.31 μm and 8.14 μm, as fine excipient materials. Inhalation formulations, each composed of a lactose carrier material, one of the fine excipient materials (2.5% or 15.0% *w*/*w*), and a drug (fluticasone propionate) material (1.5% *w*/*w*) were prepared. The physical microstructure, the rheological properties, the aerosolization pattern, and the aerodynamic performance of the formulations were studied. At low concentration, the large silica microspheres had a more beneficial influence on the drug dispersibility than the small silica microspheres. At high concentration, only the small silica microspheres had a beneficial influence on the drug dispersibility. The results reveal influences of fine excipient materials on mixing mechanics. At low concentration, the fine particles improved deaggregation and distribution of the drug particles over the surfaces of the carrier particles. The large silica microspheres were associated with a greater mixing energy and a greater improvement in the drug dispersibility than the small silica microspheres. At high concentration, the large silica microspheres kneaded the drug particles onto the surfaces of the carrier particles and thus impaired the drug dispersibility. As a critical attribute of fine excipient materials in carrier-based dry powder inhalation formulations, the particle size demands robust specification setting.

## Graphical abstract

**Figure:**
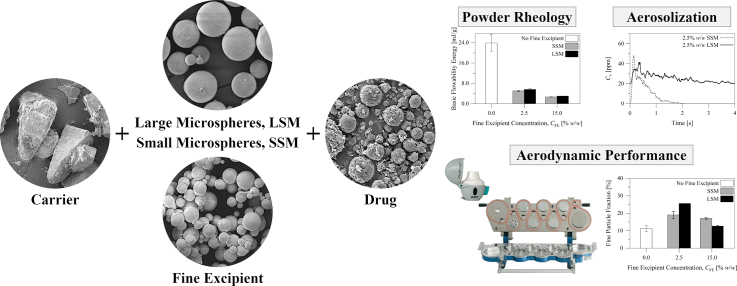

## 1 Introduction

Dry powder inhalers are a keystone in the management of respiratory diseases, such as asthma and chronic obstructive pulmonary disease. Their potential for systemic delivery of drugs and for vaccination is attracting growing interest (de Boer et al., 2017). Most dry powder inhalation (DPI) formulations in the market are carrier-based. A carrier-based DPI formulation is, typically, a blend of a coarse carrier material (particle diameter = 50–200 μm), a fine excipient material (particle diameter < 10 μm), and a respirable drug material (Elsayed and Shalash, 2018). Fine excipient particles are added to promote drug dispersibility from the formulation during inhalation. The complex nature of DPI formulations (Zeng et al., 2001) demands robust specification setting for DPI excipients.

The potential of fine excipient materials to promote drug dispersibility from carrier-based DPI formulations is well recognized (Elsayed and Shalash, 2018; Hebbink et al., 2022; Jones and Price, 2006), although it is not completely understood. Five principal mechanisms have been proposed to explain this potential. The active site theory (Grasmeijer et al., 2014a; Staniforth, 2000) suggests that fine excipient particles occupy strongly adhesive or sheltered sites on carrier particles. The agglomerate theory (Adi et al., 2006; Jones et al., 2008; Kinnunen et al., 2015; Lucas et al., 1998) suggests that fine excipient and drug particles form readily dispersible agglomerates. The fluidization enforcement hypothesis (Shur et al., 2008) suggests that fine excipient particles strengthen dispersion forces generated during aerosolization. The buffer hypothesis (Dickhoff et al., 2006; Grasmeijer et al., 2014b) suggests that fine excipient particles buffer press-on forces during mixing. The effective deagglomeration hypothesis (Shalash and Elsayed, 2017) suggests that fine excipient particles promote deaggregation of drug particles during mixing. Arguably, the potential of fine excipient materials to promote drug dispersibility from carrier-based DPI formulations involves more than one of these mechanisms. The potential depends on characteristics of the carrier material, the drug material, and the inhalation device (Hassoun et al., 2015; Hertel et al., 2018; Muddle et al., 2015).

Attempts were made to define the size of fine excipient particles dominating their contributions to drug dispersibility in carrier-based DPI formulations. Towards this goal, correlations between the concentrations of different size fractions of fine particles and the dispersibility of drug particles were studied. The correlations reported by Guenette et al. (2009) and Sun et al. (2022) suggested that the fine excipient particles are best defined as particles smaller than 8.6–12.0 μm in diameter and that these particles contribute similarly, i.e. irrespective of their definite sizes, to drug dispersibility. In contrast, the correlations reported by Handoko et al. (2009) suggested that fine lactose particles with diameters of 5–10 μm are more beneficial than those with diameters smaller than 5 μm. In agreement, Adi et al. (2006) found that a fine lactose material with a volume median particle diameter of 7.9 μm was more beneficial than a fine lactose material with a volume median particle diameter of 3.0 μm. Also in agreement, Grasmeijer et al. (2014b) found that a fine lactose material with a median particle diameter of 3.94 μm (larger than drug particles) was more beneficial than a fine lactose material with a median particle diameter of 1.95 μm (similar in size to drug particles). This could be attributed to the capability of the large fine particles to form relatively more loose agglomerates with drug particles than the small fine particles (Adi et al., 2006) and to the capability of the large fine particles to buffer press-on forces during mixing, thereby weakening adhesion of drug particles to surfaces of carrier particles (Dickhoff et al., 2006).

The aim of this study was to gain new insights into particulate interactions in carrier-based DPI formulations. To this end, we investigated the influences of the particle size of fine excipient materials on characteristics of carrier-based DPI formulations. Two particle size grades of silica microspheres were used as fine excipient materials. Silica microspheres were used to ensure narrow particle size distributions and to avoid interference from other particle characteristics, such as the shape. The influences of the shape and the surface roughness of fine excipient particles on characteristics of carrier-based DPI formulations have been recently studied and discussed by Elsayed et al. (2024). Notably, silica microspheres are not inhalation safe because of the risk of silicosis. They are used here as model fine particles. Excipient blends, each composed of a carrier material (α-lactose monohydrate) and one of the silica materials at a concentration of 2.5% or 15.0% *w*/*w*, were prepared. The two concentrations were selected, based on earlier studies (Almansour et al., 2022a; Elsayed et al., 2024), to represent two structural constructs. The low concentration illustrates, mainly, filling of macropores over the surfaces of the carrier particles and interstices between the carrier particles by silica microspheres. The high concentration illustrates separation of the carrier particles by silica microspheres. Inhalation formulations were prepared by mixing each excipient blend with an inhalable drug (fluticasone propionate) material. The influences of the two silica materials on the physical microstructure, the rheological properties, the aerosolization pattern, and the aerodynamic performance of the formulations were studied.

## 2 Materials and methods

### 2.1 Materials

The α-lactose monohydrate carrier material Inhalac® 120 (volume-weighted median diameter = 134 µm, according to the manufacturer) was kindly provided by Molkerei MEGGLE Wasserburg GmbH & Co. KG (Wasserburg am Inn, Germany). Two particle size grades of silica microspheres coated with dimethylpolysiloxane (number-weighted median diameters = 2–4 µm and 4–8 µm, according to the manufacturer) were purchased from Cospheric LLC (Santa Barbara, California, USA). Fluticasone propionate was from Jayco Chemical Industries (Maharashtra, India). Inhalable fluticasone propionate particles were prepared from the raw drug material by nano spray drying as described by Almansour et al. (2022a) and Elsayed et al. (2024). Nano Spray drying is explained in detail by Arpagaus et al. (2018) and Almansour et al. (2022b). Size-3 hypromellose capsules were kindly provided by Capsugel France S.A.S. (Colmar, France).

### 2.2 Preparation of the excipient blends and the inhalation formulations

Four excipient blends were prepared. The excipient blends were composed of the lactose carrier material (CL) with either the small silica microspheres (SSM, 2.5% or 15.0% *w*/*w*) or the large silica microspheres (LSM, 2.5% or 15.0% *w*/*w*). The excipient blends were prepared in 100-g quantities. Each 100-g blend was mixed using an IKA® EUROSTAR 20 high speed digital overhead stirrer equipped with a three-blade propeller shaft (IKA®-Werke GmbH & Co. KG, Staufen, Germany) at 400 rpm for 30 min. The rotor was 55 mm in diameter, and the mixing vessel had an internal diameter of ∼68 mm. A 100-g control sample of the carrier material was processed similarly.

Inhalation formulations were prepared via a sandwich mixing technique, as described by Almansour et al. (2022a) and Elsayed et al. (2024). Each formulation was a mixture of the inhalable drug material (fluticasone propionate, 1.5% *w*/*w*) with one of the excipient blends. The formulations were prepared in 2-g quantities. Each 2-g formulation was mixed by magnetic stirring at 60 rpm for 70 min in a 21-mL vial. The formulations were filled into Size-3 hypromellose capsules. Each capsule was filled with 25 ± 1 mg of a powder formulation, corresponding to 375 μg of the drug.

The excipient blends, the formulations, and the capsules were stored in a desiccator at room temperature for at least 3 days before characterization or evaluation to allow for mechanical relaxation and charge dissipation.

### 2.3 Characterization of the excipient and the drug materials

#### 2.3.1 Particle size distribution

Particle size distribution was measured by laser diffraction using a Malvern Mastersizer 3000 particle size analyzer, equipped with an Aero S dry dispersion (3.0 bar) unit (Malvern Instruments Ltd., Malvern, United Kingdom), as described by Elsayed et al. (2024). Mie theory of light scattering was used for analysis of laser diffraction data. The refractive index and the absorption index of α-lactose monohydrate were set to 1.521 (Shalash et al., 2015) and 0.1, respectively. The refractive index and the absorption index of silica were set to 1.457 (Kaye and Laby, 2005) and 0.01, respectively. The measurements were conducted in triplicates.

#### 2.3.2 Particle shape

Particle shape was studied by scanning electron microscopy using a ZEISS EVO LS 10 scanning electron microscope (Carl Zeiss Microscopy GmbH, Jena, Germany). For this purpose, the particles were coated with gold using a Quorum Q150R S sputter coater (Quorum Technologies Ltd., Laughton, United Kingdom) at 20 mA for 90 s.

### 2.4 Characterization of the excipient blends

#### 2.4.1 Surface coverage

Coverage of the carrier particles by the silica microspheres in each excipient blend was estimated in terms of the surface coverage ratio, SCR, as explained by Rudén et al. (2018) and Dickhoff et al. (2003). Accordingly, $\mathrm{SCR}=C_{\mathrm{FE}}S_{\mathrm{FE}}/\pi \left(100-C_{\mathrm{FE}}\right)S_{\mathrm{CE}}$, where $C_{\mathrm{FE}}$ is the concentration (% *w*/*w*) of the silica microspheres (fine excipient material) in the excipient blend, $S_{\mathrm{FE}}$ the specific surface area of the silica microspheres, and $S_{\mathrm{CE}}$ the specific surface area of the coarse carrier material. The specific surface areas were derived from the measured particle size distributions, assuming particles were spherical. For this purpose, the densities of silica and α-lactose monohydrate particles were set to 1.9 g/mL and 1.547 g/mL, respectively.

#### 2.4.2 Powder mechanics and rheology

Bulk powder characteristics (the bulk density, the compressibility, and the permeability) and dynamic powder flow characteristics (the basic flowability energy, the specific energy, the stability index, the flow rate index, and the fluidization energy) were measured using a Freeman FT4 powder rheometer (Freeman Technology, Tewkesbury, United Kingdom) as described by Elsayed et al. (2024). The measurements were conducted at least in triplicates on different powder samples.

### 2.5 Characterization and evaluation of the inhalation formulations

#### 2.5.1 Drug content uniformity

Six accurately weighed 30–60-mg samples of each formulation were dispersed in adequate volumes of a 1:1 *w*/*w* mixture of ethanol and deionized water. The dispersions were then stored overnight to allow for complete sedimentation of silica microspheres. The concentrations of the drug (fluticasone propionate) in the supernatants were then determined by UV spectrophotometry (*λ* = 240 nm), as described by Almansour et al. (2022a), using a Jenway 6715 UV/Vis. spectrophotometer (Bibby Scientific Ltd., Stone, United Kingdom).

#### 2.5.2 Physical structure

The physical microstructure of each formulation was studied by scanning electron microscopy as described in Section 2.3.2.

#### 2.5.3 Aerosolization behavior (laser diffraction)

Powder aerosolization was studied by laser diffraction using a Malvern Spraytec laser diffraction system, equipped with an inhalation cell (Malvern Instruments Ltd., Malvern, United Kingdom), as described by Elsayed et al. (2024). The formulations were aerosolized using a Handihaler® (Boehringher Ingelheim, Ingelheim, Germany), as a model inhalation device (39 L/min, 4.00 kPa, 6.2 s).

#### 2.5.4 In vitro dispersibility (cascade impaction)

The dispersibility of the drug (fluticasone propionate) particles from the formulations was studied in vitro by cascade impaction, as described by the United States Pharmacopeia (2020), using a Copley Next Generation Impactor (Copley Scientific, Nottingham, United Kingdom). The drug collection solvent was a 1:1 *w*/*w* mixture of ethanol 96% *v*/*v* and deionized water. Stages were not coated. A Handihaler® (Boehringher Ingelheim, Ingelheim, Germany) was used as a model inhalation device (39 L/min, 4.00 kPa, 6.15 s). In each experiment, five capsules were actuated, and the experiments were conducted in triplicates. Particles deposited on the capsule shells, the inhalation device, and parts and stages of the impactor were collected by rinsing with the solvent. The collected dispersions were stored overnight to allow for complete sedimentation of silica microspheres. The concentrations of the drug in the supernatants were then determined by UV spectrophotometry (*λ* = 240 nm), as described by Almansour et al. (2022a), using a Biochrom Libra S22 UV/Vis. spectrophotometer (Biochrom Ltd., Cambridge, United Kingdom). The emitted drug fraction, the fine (aerodynamic diameter < 5.0 μm) particle fraction of the emitted drug dose, and the mass median aerodynamic diameter, MMAD, of drug particles collected from the impactor stages were determined.

### 2.6 Data analysis

Data analysis was performed using OriginPro 2023 (OriginLab Corporation, Northampton, Massachusetts, USA). Presented data are always means ± standard deviations. Analysis of variance (ANOVA) with Tukey's post hoc test was used for statistical comparisons. The data reported by Elsayed et al. (2024) for excipient blends and formulations involving small silica microspheres were combined with the data measured here. Those formulations were prepared using the same materials and procedures used here.

## 3 Results

### 3.1 Characterization of the excipient materials

**Fig. 1:**
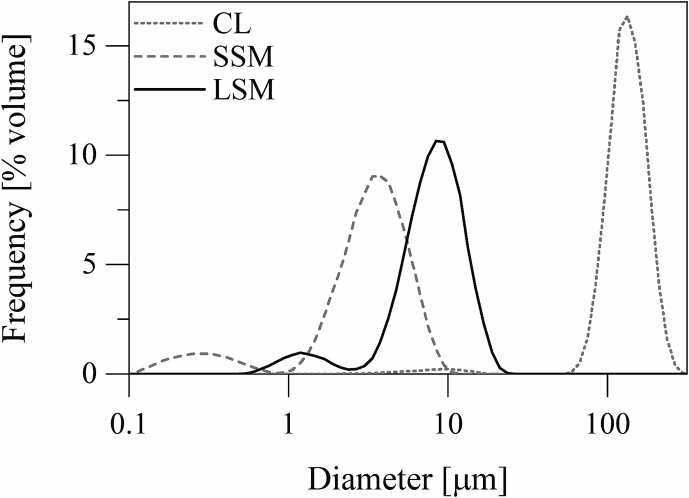
Particle size distributions of the excipient materials. CL, SSM, and LSM refer to the coarse carrier material, the small silica microspheres, and the large silica microspheres, respectively.

**Fig. 2:**
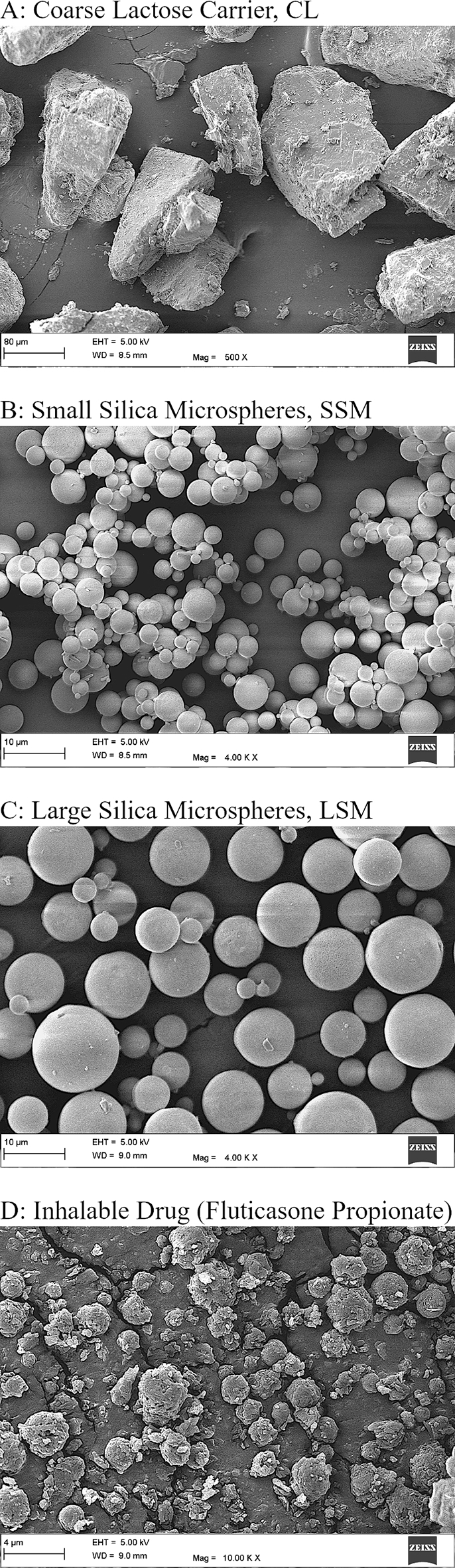
Scanning electron micrographs of A) the carrier material (scale bar length = 80 μm), B) the small silica microspheres (scale bar length = 10 μm), C) the large silica microspheres (scale bar length = 10 μm), and D) the inhalable drug material (scale bar length = 4 μm).

The particle size distributions of the excipient materials are presented in Fig. 1 and Table 1. The volume median diameter of the carrier (α-lactose monohydrate) particles was 132.60 ± 0.18 μm. The volume median diameters of the small and the large silica microspheres were 3.31 ± 0.00 μm and 8.14 ± 0.18 μm, respectively. The morphologies of the excipient particles are presented in Fig. 2. The carrier particles had the tomahawk shape characteristic of α-lactose monohydrate crystals. The small and the large silica microspheres were almost exact spheres with plain surfaces. The inhalable drug particles were quasi-spherical with rugose surfaces and were generally smaller than 5.0 μm in diameter.

**Table 1:** Particle size distributions of the excipient materials⁎*.*

Excipient Material	$D_{V,\text{Mean}}$ [μm]	$D_{V,10}$ [μm]	$D_{V,50}$ [μm]	$D_{V,90}$ [μm]	${\mathrm{FEF}}_{10.0}$ [% *v*/*v*]
Coarse Carrier (α-lactose monohydrate), CL	135.13 ± 0.36	92.07 ± 0.74	132.60 ± 0.18	185.03 ± 0.61	1.30 ± 0.25
Small Silica Microspheres, SSM	3.45 ± 0.05	0.96 ± 0.44	3.31 ± 0.00	5.91 ± 0.09	99.93 ± 0.03
Large Silica Microspheres, LSM	8.40 ± 0.15	3.99 ± 0.66	8.14 ± 0.18	13.43 ± 0.27	68.87 ± 0.60

⁎ $D_{V,\text{Mean}}$ is the volume-weighted mean particle size. $D_{V,10}$, $D_{V,50}$, and $D_{V,90}$ are the 10th percentile, the 50th percentile (the median), and the 90th percentile of the volume-weighted particle size distribution. ${\mathrm{FEF}}_{10.0}$ is the fine (D< 10.00 μm) excipient fraction. The values given are means ± standard deviations (*N* = 3).

### 3.2 Characterization of the excipient blends

Estimated surface coverage ratios, SCR, amounted to 0.43 ± 0.05 and 2.97 ± 0.32 for the excipient blends involving 2.5% and 15.0% *w*/*w* small silica microspheres (SSM), respectively, and amounted to 0.12 ± 0.01 and 0.80 ± 0.07 for the excipient blends involving 2.5% and 15.0% *w*/*w* large silica microspheres (LSM), respectively.

**Fig. 3:**
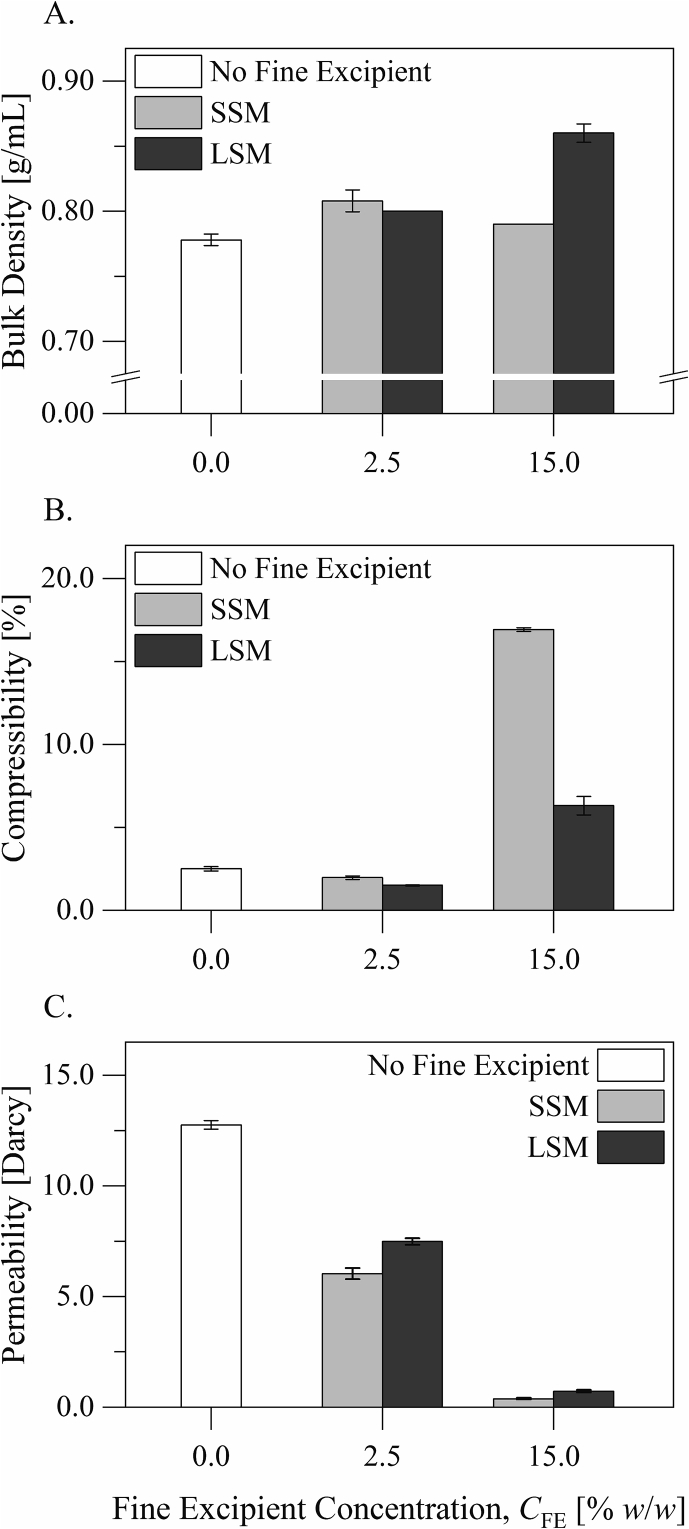
Bulk characteristics of the excipient blends: A) the bulk density, B) the compressibility under a normal stress of 15 kPa, and C) the permeability under a normal stress of 2 kPa.

Bulk characteristics of the excipient blends are presented in Fig. 3. Addition of either the small or the large silica microspheres to the carrier material to a concentration of 2.5% *w*/*w* increased the bulk density, little decreased the compressibility, and decreased the permeability of the material. These influences correspond to filling of voids between the carrier particles by silica microspheres (Almansour et al., 2022a; Elsayed et al., 2024). Addition of either the small or the large silica microspheres to the carrier material to a concentration of 15.0% *w*/*w* increased the bulk density and the compressibility of the material. The increase of the compressibility corresponds to separation of the carrier particles by the silica microspheres (Almansour et al., 2022a; Elsayed et al., 2024). The excipient blend containing the small silica microspheres at 15.0% *w*/*w* had a lower bulk density and a higher compressibility than that containing the large silica microspheres at the same concentration (*p* < 0.0001, Tukey's test following analysis of variance). This suggests that the small silica microspheres were associated with a relatively more cohesive structure than the large silica microspheres.

**Fig. 4:**
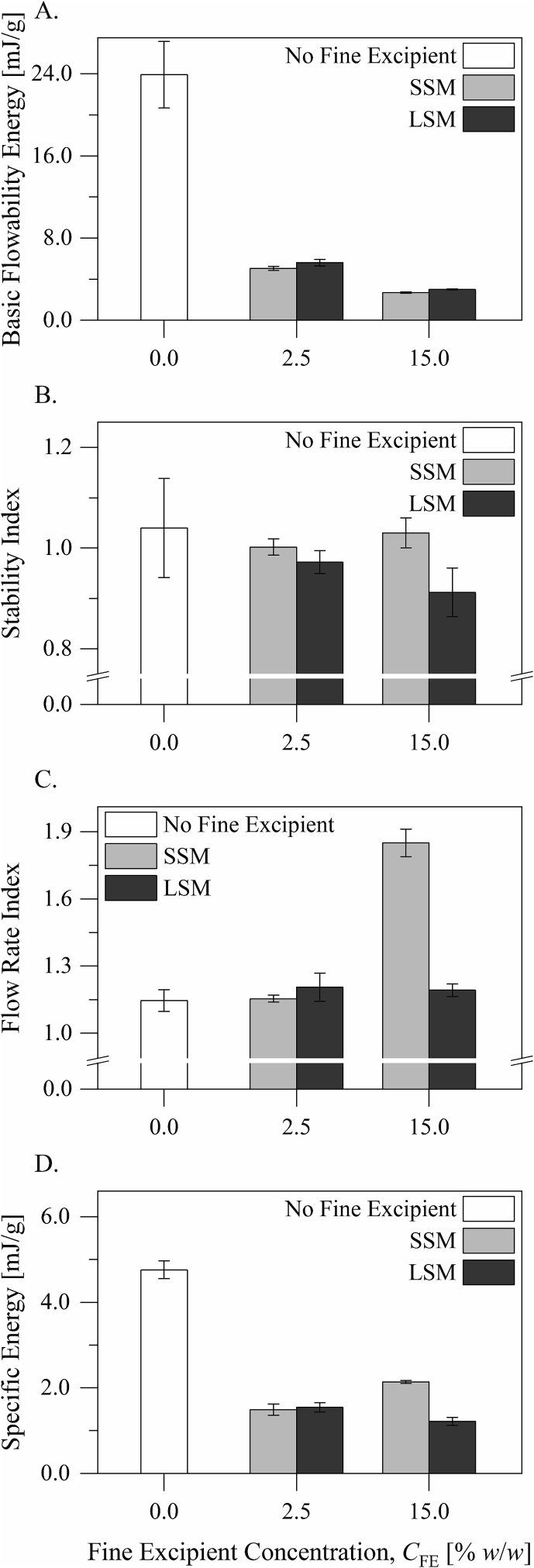
Dynamic flow characteristics of the excipient blends: A) the basic flowability evergy, B) the stability index, C) the flow rate index, and D) the specific energy.

**Fig. 5:**
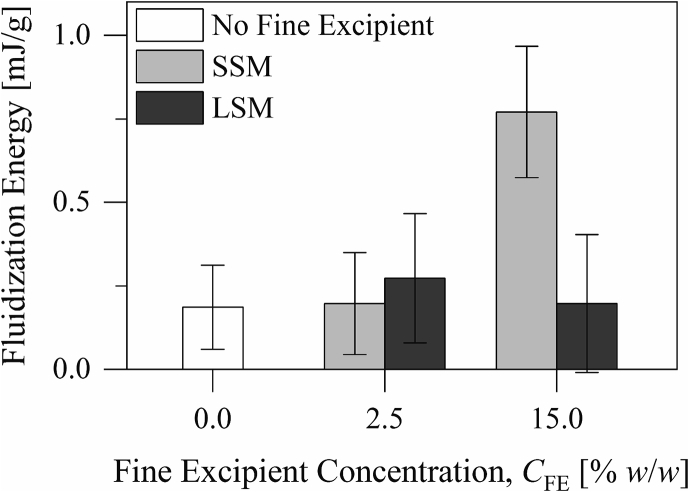
The fluidization energies of the excipient blends.

Dynamic flow characteristics of the excipient blends are presented in Fig. 4, Fig. 5. The basic flowability energy describes the powder resistance to flow during downward movement of the blade when the powder is confined; it gives prominence to the powder behavior upon consolidation. On the other hand, the specific energy describes the powder resistance to flow during upward movement of the blade when the powder is unconfined; it can reveal mechanical interlocking. The basic flowability energy and the specific energy measurements (Fig. 4A and D) suggest that the small and the large silica microspheres had similar lubricating influences on the flowability of the carrier particles. The excipient blend containing the small silica microspheres at 15.0% *w*/*w* had a higher specific energy than that containing the large silica microspheres at the same concentration (*p* < 0.0001, Tukey's test following analysis of variance). Although the specific energies of both blends were very low on the scale of the measurement, the difference suggests that the excipient blend containing the small silica microspheres was associated with a relatively greater extent of mechanical interlocking than that containing the large silica microspheres.

The stability index (Fig. 4B) describes the powder stability during repeating measurements. It is the ratio of the flowability energy measured during downward movement of the blade in the seventh test cycle to that measured in the first test cycle in a series of eleven test cycles. Only the excipient blend containing the large silica microspheres at 15.0% *w*/*w* had a stability index that was significantly smaller than 1.0 (*p* < 0.05, Student's *t*-test), suggesting that the blend had a relatively heterogeneous structure or tended to segregate upon processing. The other excipient blends and the carrier material had stability indices that were not significantly different from 1.0 (*p* > 0.05, Student's *t*-test).

The flow rate index (Fig. 4C) describes the powder sensitivity to flow rate changes. It is the ratio of the flowability energy measured during downward movement of the blade at a speed of 10 mm/s (the eleventh test cycle) to that measured at a speed of 100 mm/s (the eighth test cycle). On the other hand, the fluidization energy (Fig. 5) describes the resistance of the excipient blend to flow when fluidized. Only addition of the small silica microspheres to the carrier material to a concentration of 15.0% *w*/*w* increased the flow rate index and the fluidization energy of the material (*p* < 0.01, Tukey's test following one-way analysis of variance). It is noteworthy that the increase in the fluidization energy is much smaller than what results from addition of a micronized lactose material to the same concentration (Elsayed et al., 2024). Addition of the small or the large silica microspheres to the carrier material to a concentration of 2.5% *w*/*w* or addition of the large silica microspheres to the carrier material to a concentration of 15.0% *w*/*w* did not influence the flow rate index or the fluidization energy of the material (*p* > 0.05, Tukey's test following one-way analysis of variance). This further suggests that the small silica microspheres were associated with a relatively more cohesive structure than the large silica microspheres.

### 3.3 Characterization and evaluation of the inhalation formulations

**Fig. 6:**
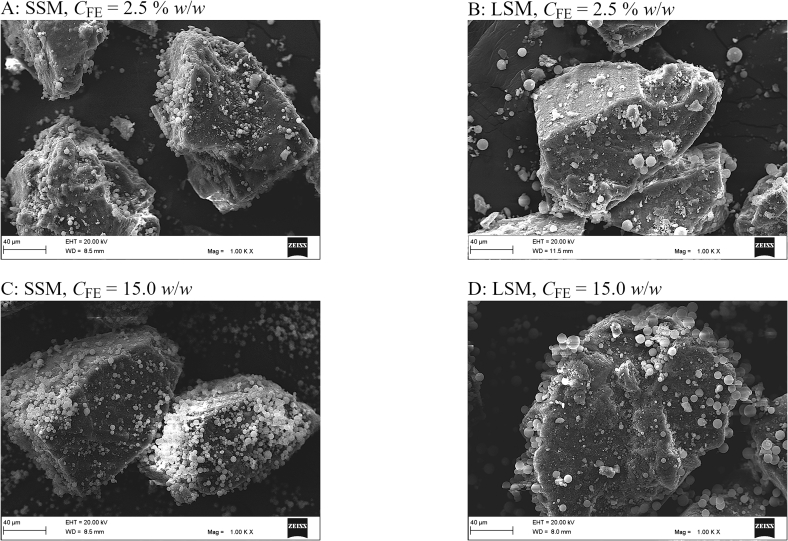
Scanning electron micrographs of the formulations involving the small silica microspheres (panels A and C) and the formulations involving the large silica microspheres (panels B and D). The scale bar at the bottom of each panel represents a length of 40 μm. The micrographs display the distribution of the particulate ingredients in the formulations.

**Fig. 7:**
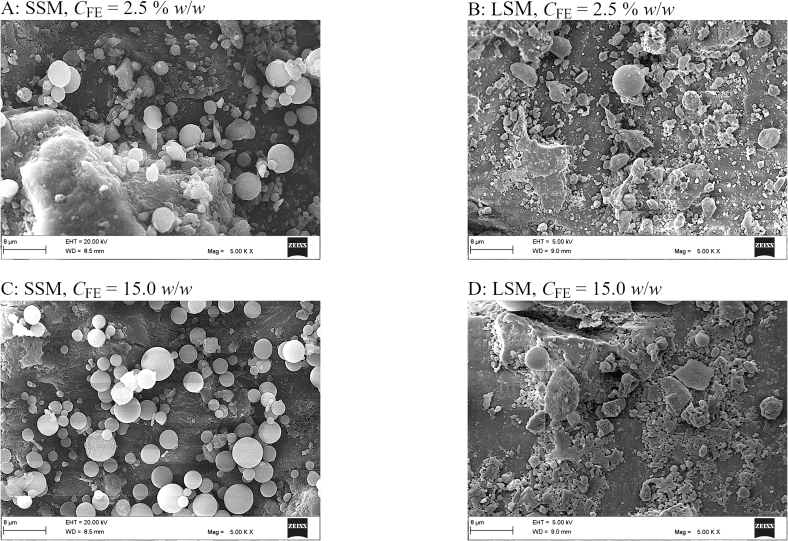
Scanning electron micrographs of the carrier particle surfaces in the formulations involving the small silica microspheres (panels A and C) and the formulations involving the large silica microspheres (panels B and D). The scale bar at the bottom of each panel represents a length of 8 μm.

The concentrations of the drug in all the formulations were satisfactorily uniform, with relative standard deviations < 5.00% (Supplementary Table S1). The Scanning electron micrographs presented in Fig. 6 display the distribution of the particulate ingredients in the formulations. Obviously, the small silica microspheres had a greater potential to aggregate and to adhere to the surfaces of the carrier particles than the large silica microspheres. The small silica microspheres indeed had a greater surface-to-volume ratio and were thus associated with greater surface coverage ratios than the large silica microspheres. Apparently, the small surface-to-volume ratio of the large silica microspheres has further limited their capacity for stable adhesion to the carrier particles. A close look at the surfaces of the carrier particles (Fig. 7) reveals another difference between the formulations. In the formulations involving the small silica microspheres, the carrier particles appeared covered by distinguishable and intact silica microspheres and drug particles. On the other hand, in the formulations involving the large silica microspheres, the carrier particles appeared covered by compressed, ground fine particle fragments. It was not clear whether the fine fragments were fragments of the carrier particles or the drug particles. However, it was obvious that the particles in the formulations involving the large silica microspheres were subject to milling. The weak adhesion of the large silica microspheres to the carrier particles can offer a possible explanation for this observation. The large silica microspheres may have behaved like balls in a ball mill. During mixing, the relatively free movement of the large silica microspheres, which were also heavier than the small silica microspheres, may have led to fragmentation of the edges of the carrier particles and/or milling of the drug particles.

**Fig. 8:**
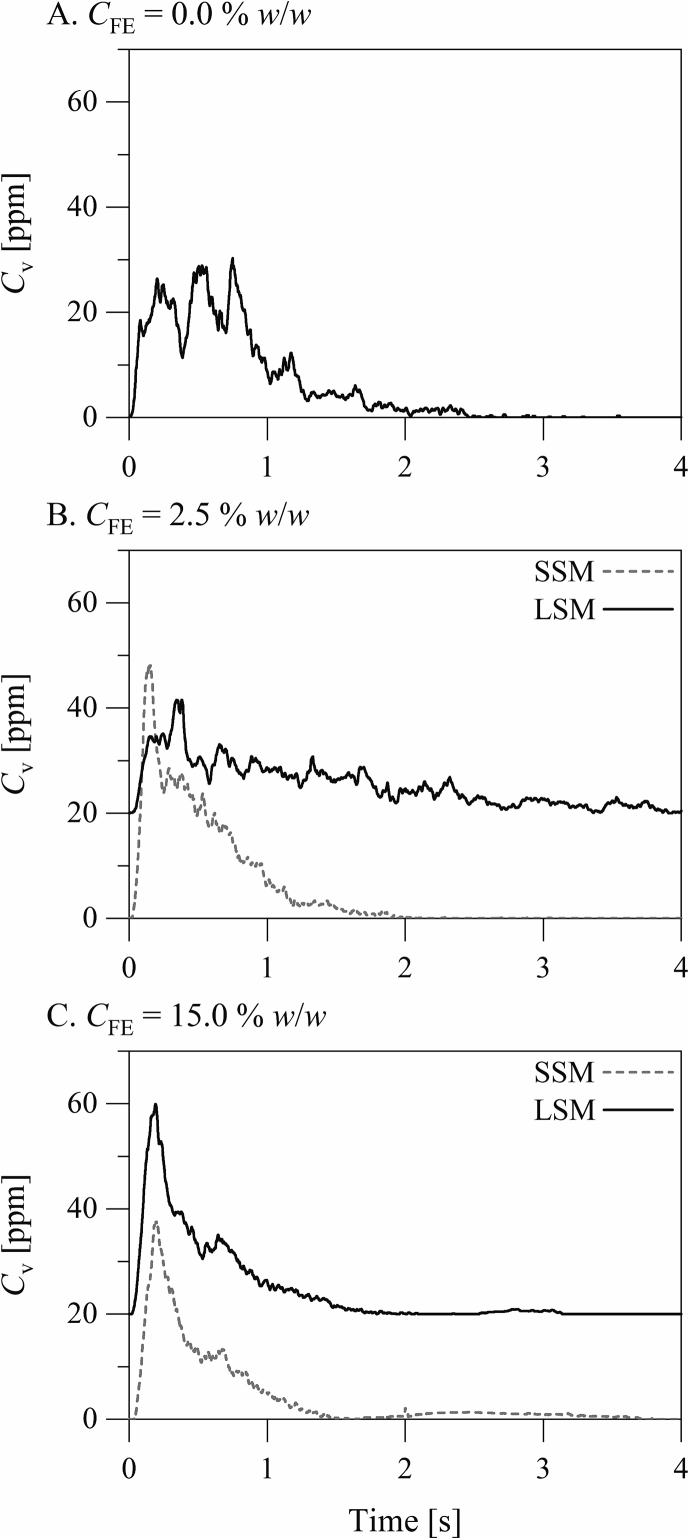
The aerosolization profiles of the inhalation formulations. The aerosolization profiles are plots of the concentration (ppm *v*/*v*) of particles entrained in the air stream after actuation versus time. The presented profiles are averages of replicate measurements. The data of the formulations involving the large silica microspheres are offset by 20 ppm *v*/*v*.

**Fig. 9:**
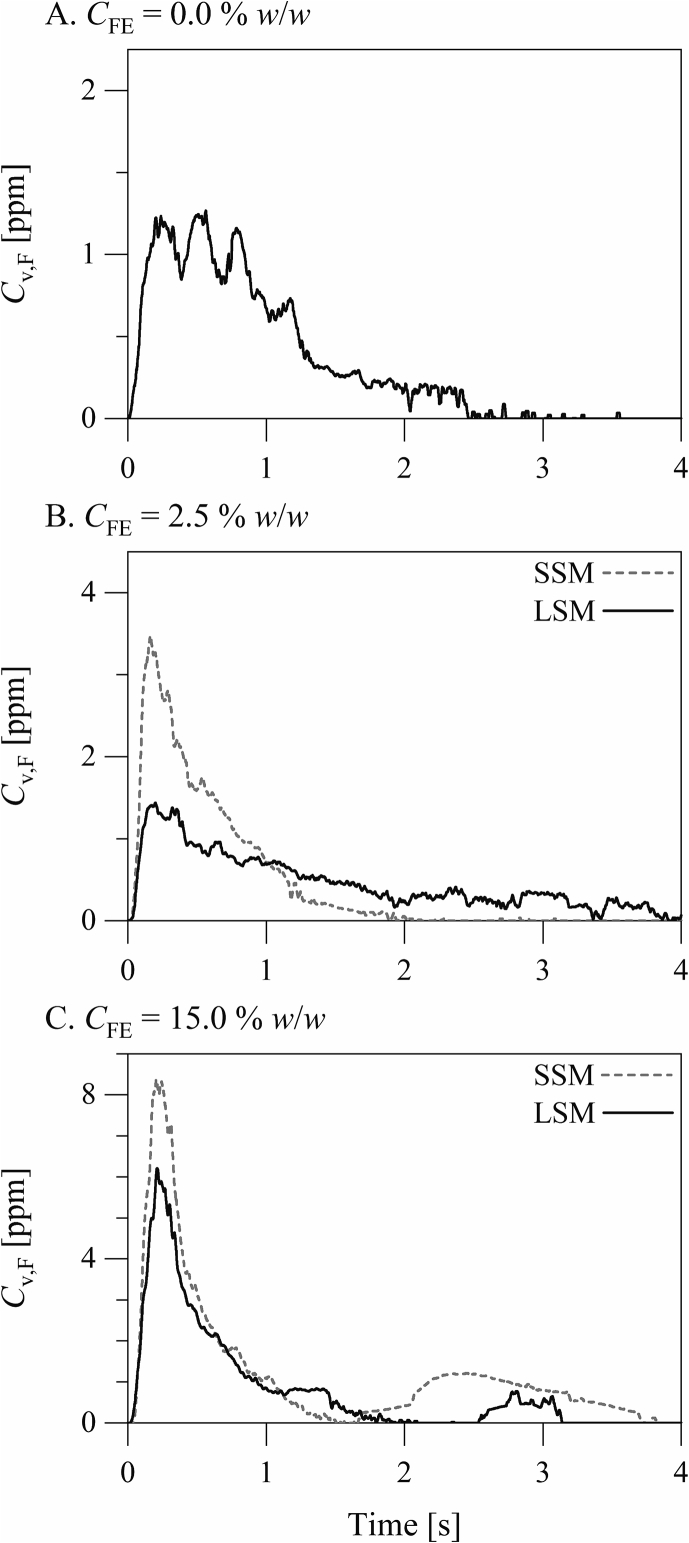
The fine particle dispersion profiles of the inhalation formulations. The fine particle dispersion profiles are plots of the concentration (ppm *v*/*v*) of fine (D < 20 μm) particles entrained in the air stream after actuation versus time. The presented profiles are averages of replicate measurements.

The aerosolization profiles of the inhalation formulations are presented in Fig. 8. The aerosolization profiles, which are measured by laser diffraction, are plots of the concentration of particles entrained in the air stream after actuation versus time. Aerosolization of the formulation containing no silica microspheres took place in the form of multiple similar bursts, suggesting that the aerosolization took place by erosion or mild fracture. Silica microspheres at $C_{\mathrm{FE}}=$ 2.5% *w*/*w* ($C_{\mathrm{FE}}$ is the fine material concentration in the excipient blend) had a considerable influence on the mechanism of aerosolization. Aerosolization of the formulation containing the small silica microspheres at $C_{\mathrm{FE}}=$ 2.5% *w*/*w* took place in the form of an initial burst followed by a decaying stream. This suggests that the aerosolization took place by fracture. On the other hand, aerosolization of the formulation containing the large silica microspheres at $C_{\mathrm{FE}}=$ 2.5% *w*/*w* took place in the form of a relatively uniform stream over approximately 4 s. This suggests that the aerosolization took place by slow erosion. The aerosolization profiles of the formulations containing either the small or the large silica microspheres at $C_{\mathrm{FE}}=$ 15.0% *w*/*w* suggest a similar fracture mechanism. The fine particle dispersion profiles of the inhalation formulations are presented in Fig. 9. These are plots of the concentration of fine (D < 20 μm) particles entrained in the air stream after actuation versus time. Fine particles are here defined as particles smaller than 20 μm in diameter in order account completely for the small the large silica microspheres. Notably, the aerosolization profiles measured by laser diffraction do not discriminate between fine excipient and drug particles. The fine particle dispersion profiles and the gross aerosolization profiles were similar, suggesting that fine (drug and silica) and coarse (carrier) particles were released from each formulation generally concomitantly. The formulations containing either the small or the large silica microspheres at $C_{\mathrm{FE}}=$ 15.0% *w*/*w* exhibited a second, discrete stream of fine particles after the decay of the main stream. The amount of fine particles released, estimated from the area under the curve of the fine particle dispersion profile, was obviously greater from the formulations containing the small silica microspheres at $C_{\mathrm{FE}}=$ 15.0% *w*/*w* than that containing the large silica microspheres at the same concentration.

**Fig. 10:**
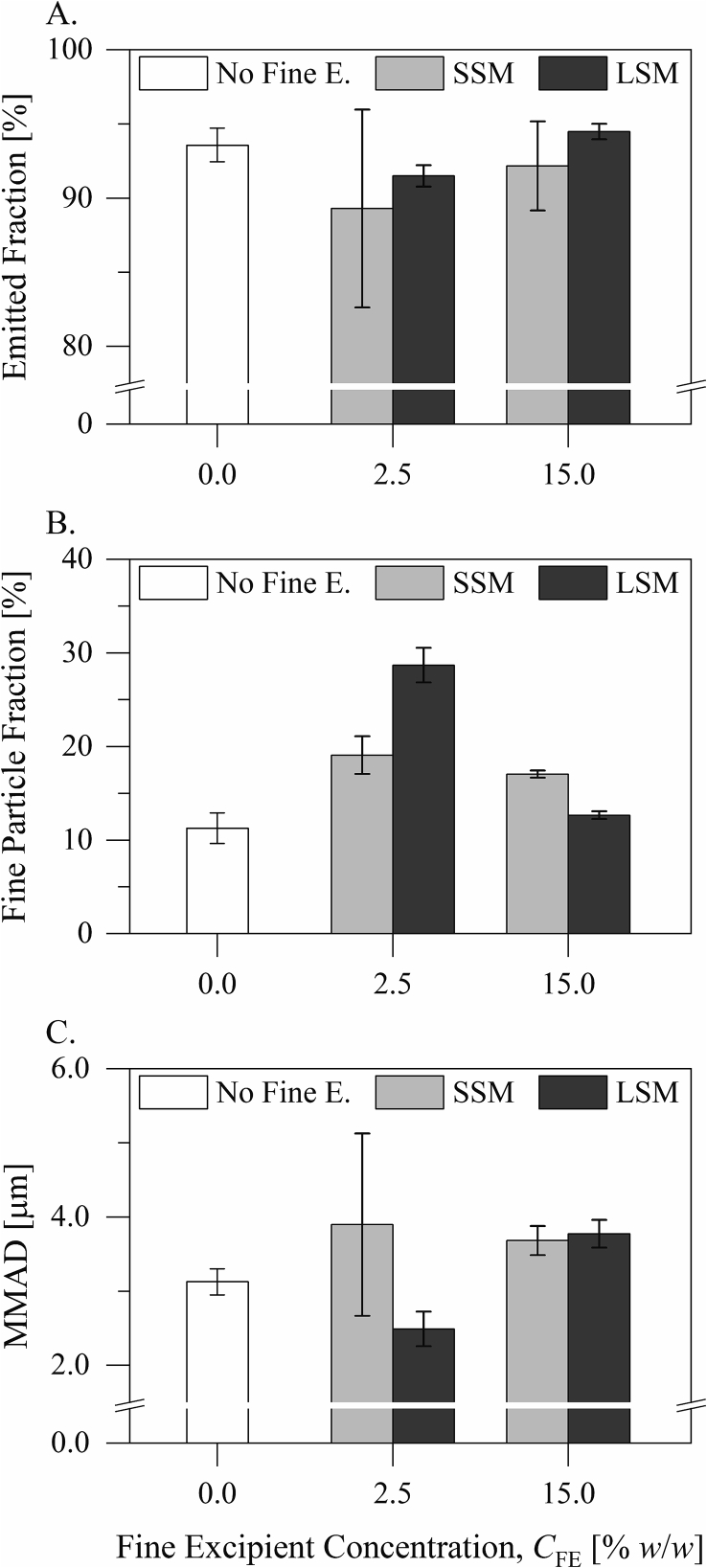
The aerodynamic performance of the inhalation formulations: A) the emitted drug fraction, B) the fine particle fraction of the emitted drug dose, and C) the mass median aerodynamic diameter (MMAD) of the dispersed drug particles.

The aerodynamic performance of the inhalation formulations is presented in Fig. 10 and in Supplementary Fig. S1. The formulations exhibited statistically similar emitted drug fractions (*p* > 0.05, one-way analysis of variance and Tukey's test), which generally amounted to 92.20% ± 3.37% *w*/*w*. The formulations containing the small or the large silica microspheres at $C_{\mathrm{FE}}=$ 2.5% *w*/*w* exhibited greater fine drug particle fractions than the formulation containing no silica microspheres (*p* < 0.001, Tukey's test following one-way analysis of variance). The formulation containing the large silica microspheres at $C_{\mathrm{FE}}=$ 2.5% *w*/*w* exhibited a greater fine drug particle fraction than that containing the small silica microspheres at the same concentration (28.68% ± 1.85% vs 19.07% ± 2.02% *w*/*w*, *p* < 0.0001, Tukey's test). Increasing the concentration of the small silica microspheres from $C_{\mathrm{FE}}=$ 2.5% *w*/*w* to $C_{\mathrm{FE}}=$ 15.0% *w*/*w* did not have a significant effect on the drug dispersibility (*p* > 0.05, Tukey's test). However, increasing the concentration of the large silica microspheres from $C_{\mathrm{FE}}=$ 2.5% *w*/*w* to $C_{\mathrm{FE}}=$ 15.0% *w*/*w* decreased the drug dispersibility (*p* < 0.0001, Tukey's test). The formulation containing the large silica microspheres at $C_{\mathrm{FE}}=$ 15.0% *w*/*w* exhibited a smaller fine drug particle fraction than that containing the small silica microspheres at the same concentration (12.64% ± 0.41% vs 17.04% ± 0.39% *w*/*w*, *p* < 0.05, Tukey's test). The mass median aerodynamic diameters of the drug particles collected from the impactor stages were statistically similar from all the formulations (3.39 ± 0.73 μm, *p* > 0.05, one-way analysis of variance and Tukey's test), despite that the dispersed drug particles were apparently smaller from the formulation containing the large silica microspheres at $C_{\mathrm{FE}}=$ 2.5% *w*/*w* than from the other formulations.

## 4 Discussion

In this study, the influences of the particle size of fine excipient materials on various characteristics of carrier-based DPI formulations were investigated. Silica microspheres were used as fine excipient materials to ensure narrow particle size distributions and to avoid interference from other particle characteristics. The shape and the surface roughness of fine particles in carrier-based DPI formulations can affect the contributions of the particles to the dispersibility of the formulations (Elsayed et al., 2024; Rudén et al., 2021). Two particle size grades of silica microspheres, with volume-weighted median diameters of 3.31 ± 0.00 μm and 8.14 ± 0.18 μm, were studied. Two concentrations of each size grade of silica microspheres were studied. The concentration ($C_{\mathrm{FE}}$, the fine material concentration in the carrier-fine material excipient blend) of 2.5% *w*/*w* corresponded to surface coverage ratios, SCR, of 0.43 ± 0.05 and 0.12 ± 0.01 for the excipient blend involving the small silica microspheres (SSM) and that involving the large silica microspheres (LSM), respectively. This concentration illustrates, mainly, filling of macropores over the surfaces of the carrier particles and interstices between the carrier particles by silica microspheres. On the other hand, the concentration of 15.0% *w*/*w* corresponded to surface coverage ratios, SCR, of 2.97 ± 0.32 and 0.80 ± 0.07 for the excipient blend involving the small silica microspheres (SSM) and that involving the large silica microspheres (LSM), respectively. This concentration illustrates separation of the carrier particles by silica microspheres.

The small and the large silica microspheres had different influences on the mechanism of powder aerosolization (Fig. 8, Fig. 9). The small silica microspheres shifted the mechanism of aerosolization from erosion or mild fracture towards severe fracture. This agrees with earlier observations (Shur et al., 2008; Tuley et al., 2008; Watling et al., 2010), showing that the severity of fracture of powder materials during aerosolization increases with the concentration of fine particles. The cohesivity imparted by fine particles enhances the capacity of the powder material to withstand dispersion forces and shifts the mechanism of aerosolization of the powder material from mild erosion towards severe fracture. The influence of the large silica microspheres on the mechanism of powder aerosolization was different and was concentration dependent. The formulation containing the large silica microspheres at $C_{\mathrm{FE}}=$ 2.5% *w*/*w* ($C_{\mathrm{FE}}$ is the fine material concentration in the excipient blend) was aerosolized by extended erosion. On the other hand, the formulation containing the large silica microspheres at $C_{\mathrm{FE}}=$ 15.0% *w*/*w* was aerosolized by fracture, similar to the formulations containing the small silica microspheres. This suggests that the large silica microspheres at $C_{\mathrm{FE}}=$ 2.5% *w*/*w* enhanced the powder flow but at $C_{\mathrm{FE}}=$ 15.0% *w*/*w* increased the powder cohesivity.

The small and the large silica microspheres also had different influences on the drug dispersibility from the formulations (Fig. 10). At $C_{\mathrm{FE}}=$ 2.5% *w*/*w*, the large silica microspheres had a more beneficial influence on the drug dispersibility than the small silica microspheres. This agrees with the observations reported by Adi et al. (2006) and Grasmeijer et al. (2014b) and can be attributed to the capacity of the larger fine particles to form relatively more loose agglomerates (Adi et al., 2006) and to buffer press-on forces during mixing (Dickhoff et al., 2006). However, only the small silica microspheres had here a beneficial influence on the drug dispersibility at $C_{\mathrm{FE}}=$ 15.0% *w*/*w*. This cannot be explained by the agglomerate and the buffer hypotheses. The scanning electron micrographs of the formulations (Fig. 6, Fig. 7) point to particulate interactions in the mixing process.

Mixing is a key process in the production of carrier-based DPI formulations (Grasmeijer et al., 2013; Jones et al., 2010; Kaialy, 2016). Considering the contributions of the fine excipient particles to the mixing process can explain the influences of the particle size of the silica microspheres on the drug dispersibility. Mixing of carrier-based DPI formulations involve four processes: i) random mixing, ii) deaggregation of fine particles, iii) adhesion of fine particles to carrier particles, and iv) redistribution and compression of fine particles onto the surfaces of carrier particles (Nguyen et al., 2015). The influences of the mixing process on drug dispersibility from carrier-based DPI formulations can be illustrated by the mixing energy concept, developed by Thalberg and colleagues (Thalberg et al., 2024a; Thalberg et al., 2024b; Thalberg et al., 2021). Accordingly, drug dispersibility from carrier-based DPI formulations could be expressed as a product of two exponential terms, which are both functions of the mixing energy. The first term accounts for an initial increase in drug dispersibility, whereas the second term accounts for a decrease in drug dispersibility upon extensive mixing. The mixing energy expressions suggested by Thalberg et al. (2021) did not account for the mass of ternary fine ingredients since the concept was applied to systems involving ternary ingredients which were assumed to act mainly as coating agents. Silica microspheres did not here behave as coating agents but rather as mixing aids. Plausibly, the mixing energy here was a function of the fine particle size (i.e., mass) and concentration. At the low concentration, the silica microspheres improved deaggregation and distribution of the drug particles over the surfaces of the carrier particles (Shalash and Elsayed, 2017). The large silica microspheres were associated with greater improvement in drug dispersibility than the small silica microspheres (the first term). Upon increasing the concentration of the silica microspheres, the large silica microspheres were associated with considerable kneading of the drug particles onto the surfaces of the carrier particles and thus a remarkable decline in the drug dispersibility (the second term).

The data presented here highlight two roles of fine excipient materials in carrier-based DPI formulations: the contributions to the mechanism of aerosolization and the contributions to the mixing process. Both contributions appear to be particle size dependent and concentration dependent. The use of silica microspheres here has probably emphasized these contributions. With other fine excipient materials, such as micronized lactose materials with irregular particle shape, other contributions, such as the contributions to saturation of active sites (Grasmeijer et al., 2014a; Staniforth, 2000), the contributions to formation of agglomerates with drug particles (Adi et al., 2006; Jones et al., 2008; Lucas et al., 1998), and the contributions to fluidization enforcement (Shur et al., 2008), potentially dominate. The data offer a better understanding of different influences of different ternary fine excipient materials and force control agents. The data warrant a need for robust specification setting. Small differences in the size of fine excipient particles can have considerable influences on the performance of carrier-based DPI formulation. The data also warrant a need for robust setting of particle size characterization methods. For example, Mie theory of light scattering rather than Fraunhofer approximation should be used to analyze laser diffraction measurements of DPI materials (Elsayed, 2019).

## 5 Conclusions

The particle size is a critical attribute of fine excipient materials in carrier-based dry powder inhalation formulations. It demands robust specification setting and high-resolution particle size characterization methods. The effects of the particle size of fine excipient materials on the performance of carrier-based dry powder inhalation formulations are concentration dependent and are potentially modulated by other characteristics of the fine excipient particles, the carrier particles, and the drug particles. The contributions of fine excipient materials in carrier-based DPI formulations to the mixing process are warranted.

## CRediT authorship contribution statement

**Mustafa M.A. Elsayed:** Conceptualization, Funding acquisition, Project administration, Methodology, Investigation, Formal analysis, Visualization, Writing – original draft. **Iman M. Alfagih:** Methodology, Validation, Investigation. **Katrina Brockbank:** Methodology, Validation, Investigation, Writing – review & editing. **Fawaz Alheibshy:** Validation, Investigation. **Alhassan H. Aodah:** Validation, Investigation. **Raisuddin Ali:** Methodology, Investigation, Visualization. **Khaled Almansour:** Validation, Investigation. **Ahmed O. Shalash:** Methodology, Formal analysis, Writing – review & editing.

## Declaration of competing interest

The authors declare that they have no known competing financial interests or personal relationships that could have appeared to influence the work reported in this paper. The funders had no role in the study design, in the collection, analysis and interpretation of data, in the writing of the manuscript, or in the decision to submit the article for publication.

## Data Availability

Data will be made available on request.

## Appendix A Supplementary data

Supplementary material Image 1
