# Supplementary material for: Fine excipient materials in carrier-based dry powder inhalation formulations: The interplay of particle size and concentration effects
Source: Int J Pharm X. 2024 May 1;7:100251. doi: 10.1016/j.ijpx.2024.100251 (PMC11127535; doi:10.1016/j.ijpx.2024.100251)
Supplement: Supplementary file 1 — Supplementary material [file mmc1.pdf]

Fine excipient materials in carrier-based dry powder inhalation formulations: The interplay of particle size and concentration effects

Mustafa M.A. Elsayed [a,b,*,1], Iman M. Alfagih [c], Katrina Brockbank [d], Fawaz Alheibshy [a], Alhassan H. Aodah [e], Raisuddin Ali [c], Khaled Almansour [a], Ahmed O. Shalash [f]

[a] *Department of Pharmaceutics, College of Pharmacy, University of Ha'il, Ha'il, Saudi Arabia*
[b] *Department of Pharmaceutics, Faculty of Pharmacy, Alexandria University, Alexandria, Egyptg*
[c] *Department of Pharmaceutics, College of Pharmacy, King Saud University, Riyadh, Saudi Arabia*
[d] *Freeman Technology Ltd., Tewkesbury, United Kingdom*
[e] *Advanced Diagnostics and Therapeutics Institute, Health Sector, King Abdulaziz City for Science and Technology (KACST), Riyadh, Saudi Arabia*
[f] *School of Chemistry and Molecular Biosciences, The University of Queensland, St. Lucia, Queensland, Australia*

Supplementary Data

Table S1: Drug contents in the inhalation formulations

Formulation	Drug Concentration [% *w*/*w*]
No Fine Excipient Material	1.45 ± 0.04
SSM, C_{FE} = 2.5 % *w*/*w*	1.37 ± 0.04
LSM, C_{FE} = 2.5 % *w*/*w*	1.42 ± 0.02
SSM, C_{FE} = 15.0 % *w*/*w*	1.56 ± 0.07
LSM, C_{FE} = 15.0 % *w*/*w*	1.50 ± 0.04

[*] The values given are means ± standard deviations (N = 6).

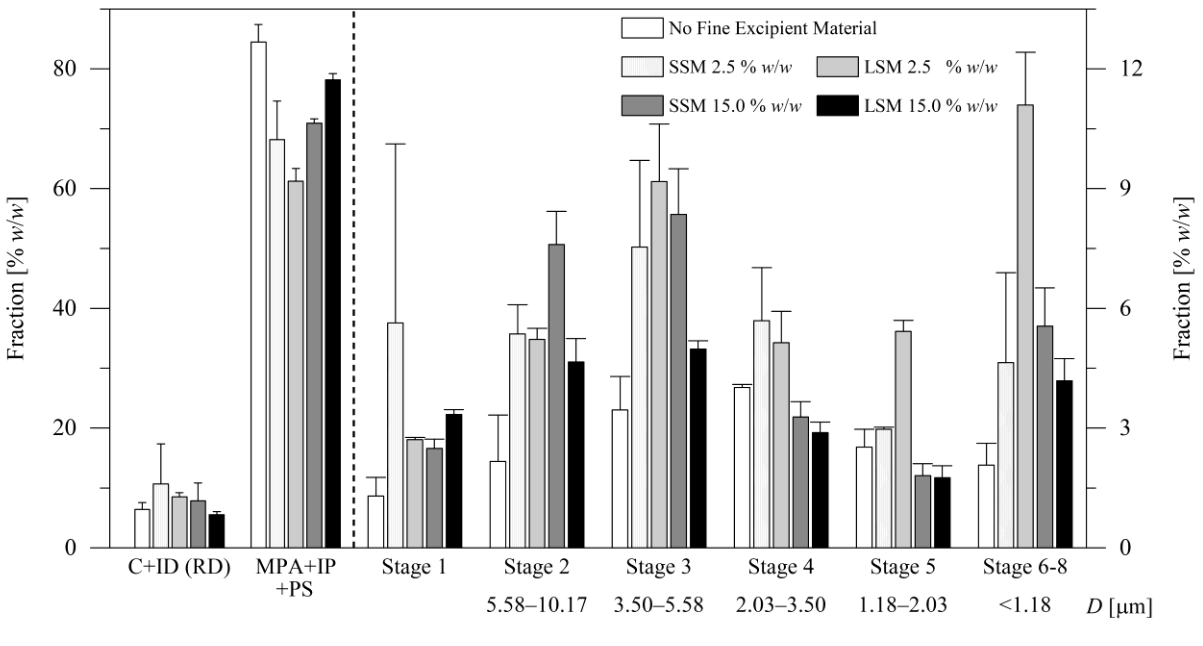

Fig. S1. Drug dispersibility from the inhalation formulations as measured by cascade impaction. C+ID refers to the drug collected from the capsule shells and the inhalation device and is presented as a fraction of the recovered dose (left axis). MPA+IP+PS refers to the drug collected from the induction port, the mouthpiece adapter, and the preseparator and is presented as a fraction of the emitted dose (left axis). The drug collected from each of the impactor stages is presented as a fraction of the emitted dose (right axis). The size range of particles collected on each of the impactor stages is given.
